# Maximal Extraction of Biological Information from Genetic Interaction Data

**DOI:** 10.1371/journal.pcbi.1000347

**Published:** 2009-04-03

**Authors:** Gregory W. Carter, David J. Galas, Timothy Galitski

**Affiliations:** Institute for Systems Biology, Seattle, Washington, United States of America; Johns Hopkins University, United States of America

## Abstract

Extraction of all the biological information inherent in large-scale genetic interaction datasets remains a significant challenge for systems biology. The core problem is essentially that of classification of the relationships among phenotypes of mutant strains into biologically informative “rules” of gene interaction. Geneticists have determined such classifications based on insights from biological examples, but it is not clear that there is a systematic, unsupervised way to extract this information. In this paper we describe such a method that depends on maximizing a previously described context-dependent information measure to obtain maximally informative biological networks. We have successfully validated this method on two examples from yeast by demonstrating that more biological information is obtained when analysis is guided by this information measure. The context-dependent information measure is a function only of phenotype data and a set of interaction rules, involving no prior biological knowledge. Analysis of the resulting networks reveals that the most biologically informative networks are those with the greatest context-dependent information scores. We propose that these high-complexity networks reveal genetic architecture at a modular level, in contrast to classical genetic interaction rules that order genes in pathways. We suggest that our analysis represents a powerful, data-driven, and general approach to genetic interaction analysis, with particular potential in the study of mammalian systems in which interactions are complex and gene annotation data are sparse.

## Author Summary

Targeted genetic perturbation is a powerful tool for inferring gene function in model organisms. Functional relationships between genes can be inferred by observing the effects of multiple genetic perturbations in a single strain. The study of these relationships, generally referred to as genetic interactions, is a classic technique for ordering genes in pathways, thereby revealing genetic organization and gene-to-gene information flow. Genetic interaction screens are now being carried out in high-throughput experiments involving tens or hundreds of genes. These data sets have the potential to reveal genetic organization on a large scale, and require computational techniques that best reveal this organization. In this paper, we use a complexity metric based in information theory to determine the maximally informative network given a set of genetic interaction data. We find that networks with high complexity scores yield the most biological information in terms of (i) specific associations between genes and biological functions, and (ii) mapping modules of co-functional genes. This information-based approach is an automated, unsupervised classification of the biological rules underlying observed genetic interactions. It might have particular potential in genetic studies in which interactions are complex and prior gene annotation data are sparse.

## Introduction

Understanding the functional interactions between genes remains a major challenge in the genetics of complex traits and a fundamental problem in biology. Organisms can be viewed as information processors, highly evolved to recognize environmental conditions and respond in a way that maximizes fitness. A major goal of systems biology is to understand these processes by perturbing system elements, assaying the resulting phenotypes, and modeling system responses [1]. These phenotypes are determined by complex interactions among gene variants and environmental factors. Thus, the classical genetics approach of combining perturbations to infer functional relationships between genes (genetic interactions) can provide especially useful information for system modeling. In model organisms, particularly in yeast, such experiments have been performed systematically and at high throughput [2]–[7]. In outbred populations, advances in QTL mapping allow identification of multiple trait loci [8]–[11]. Extracting the information from these data sets that best describes the hidden organization of gene activity is a major challenge that we address here.

Detecting and classifying genetic interactions in a way that maximizes the information content of the data should correspond to the most biologically informative mapping of relationships between genes. This idea casts the problem in terms of information classification, rendering it a computational challenge for genetic interaction data involving many genes. This classification problem has been approached at various levels of analytic detail. Tong and collaborators [2] considered only one type of interaction (synthetic growth defects), generating a network of binary edges between genetic knockouts (interacting or not interacting). Zhong and Sternberg [12] also classified genetic interaction in binary terms, as any genetic non-independence or genetic independence. The analysis of Segré, et al. and others [6],[13],[14] adds a level of detail by classifying genetic interactions as aggravating, alleviating, or neutral, with later studies further sub-classifying the alleviating interactions [6]. The work of Drees, et al. [5] segregates the interactions into nine classes, chosen to best correspond to interaction types identified as being useful in classical genetic studies. For all of these approaches, it is possible that alternative classification schemes may reveal more biological information in the same data, since unexpected but meaningful relationships may be missed when not explicitly sought. As genetic-interaction experiments are performed on larger scales, it will become increasingly difficult to preconceive the most informative classification. This is the problem then, to extract the maximum information from a given data set by optimizing the classification of interactions. We call the interaction classes ***rules*** in this paper.

The classification problem is apparent when the classical genetics strategy of using genetic interactions to order pairs of genes in a biochemical pathway is applied to a large-scale data set. For example, classical epistasis occurs between two mutant alleles with different phenotypes, when the double-mutant phenotype is the same as that of one of the single mutations (the one bearing the epistatic mutation), and the other (hypostatic) mutation is thereby masked (The term epistasis is sometimes used to as a generic term meaning any genetic interaction. Here we use it in the narrow sense of one mutant completely masking the effect of another.). The standard, pathway interpretation of this interaction is serial information flow from one gene to the other gene to the phenotype [15]. A serial model, however, could also predict a different genetic interaction in which both single-mutants and the double-mutant have identical (non-wild-type) phenotypes, particularly in cases in which the upstream gene activates the downstream gene. This type of interaction is sometimes called “complementary epistasis” [16] or “asynthesis” [5]. Both epistasis and asynthesis suggest a model with serial regulation of the phenotype, and it is not clear when these two types of serial regulation encode an informative distinction when embedded in a large-scale data set. Such issues of interpretation are compounded by differences in the directions of phenotype effects, which can be positive or negative, and variations in mutation type, since gain-of-function and loss-of-function mutants will often have different interpretations. Thus it is unclear how biologically meaningful the distinctions between interactions are in a given data set, and the meaning will likely depend on the function of the biological system and genes being studied.

It is therefore not surprising that classical pathway reconstruction techniques that have proven successful for interpreting the relationships between isolated gene pairs have not been directly scalable to global interaction networks. It is likely that a different type of information on genetic architecture might be obtained when the data are subjected to an alternate analysis and interpretation. In particular, large-scale data sets involving interactions among many genes might reveal an underlying organization in terms of modules of co-functional genes. This organization may be missed by pathway analysis developed for preselected gene pairs. A model-independent, agnostic and data-driven approach is necessary to clarify this issue and generate networks from which the most information can be extracted.

We address this question here by proposing that the most biologically informative classification scheme is the one that leads to the greatest amount of biological complexity accounted for in the set of interacting genes. We use the term complexity here somewhat differently than is usual. We propose to maximize the complexity in the set according to a previously described complexity measure that is context-dependent, discounts both redundancy and randomness, and depends on the entire set of genes [17]. Finding the classification that gives us the greatest information content requires: (i) a systematic encoding of genetic interaction data, (ii) a metric for measuring the complexity of a given classification scheme. To encode genetic interactions, we follow the strategy of Drees, et al. [5]. To measure the complexity we use the context-dependent complexity metric developed in our previous work [17] on networks constructed from given data sets and multiple classification schemes.

The complexity metric we use depends *only* on the genetic interaction network generated by a given data set and classification scheme. Although network topology (the set of nodes and the presence or absence of an edge between each node pair) is fixed for each data set by experimental design (the set of genetic perturbations used and the pairs tested), edge types vary according to the classification scheme used and thereby alter the network. To test the biological relevance of a given classification scheme [15], we assess the resulting network for local enrichment of interaction with genes of a common biological function and search for clusters of genes with similar interaction patterns across the global network. We find that these clusters correspond to high-order functional organization, or modules [5]. A biologically informative network is expected to encode both localized functional enrichments and dense modules, whereas a non-informative network will produce few enrichments and map sparse modules.

Although the terms information, complexity, and entropy are often used synonymously in the literature, from this point forward in this work we will use the term *complexity* to mean the numerical quantity calculated according to our measure for a given interaction network (Ψ, as defined below). By optimizing on this complexity score, we can determine the maximally informative classification and study the corresponding genetic interaction network for biological relevance.

## Results

### Systematic quantification of genetic interactions

Genetic interactions are defined for a chosen phenotype under specific environmental conditions, so that the only variables are the specific genetic perturbations carried by the strains being compared. This allows the modes of genetic interaction to be systematically analyzed and formally classified [5]. Consider a genotype *X*, and its cognate observed phenotype, *P_X_*. The phenotype could be a quantitative measurement or any other observation that can be clearly compared across mutant genotypes (e.g. slow vs. standard vs. fast growth, color or shape of colony, invasiveness of growth on agar etc.). The genotype is usually labeled by the mutation of one or more genes, which could be gene deletions, high-copy amplifications, or other allele forms. With genotypes labeled by mutant alleles, a set of four phenotype observations can be assembled which defines a genetic interaction: *P_A_* and *P_B_* for gene A and gene B mutant alleles, *P_AB_* for the AB double mutant, and *P_WT_* for the wild type or reference genotype. The relationship among these four measurements defines a genetic interaction; for example, if we follow the classic genetic definitions described above, *P_AB_ = P_A_<P_WT_<P_B_* describes one type of epistatic interaction, while *P_WT_<P_AB_ = P_A_ = P_B_* is an example of asynthesis. There are a total of 45 distinct inequalities that can be constructed from four phenotypes [5], and these are the interactions we need to classify.

We consider two genetic interaction data sets from the literature that use yeast as a model system (Materials and Methods). The first is a study of genetic interactions observed in invasive growth [5]. When exposed to low-nitrogen growth conditions, the *Saccharomyces cerevisiae* laboratory strain Σ1278b undergoes a cell differentiation from round single-cell growth to a pathogen-like filamentous form marked by cell elongation, unipolar distal budding, and invasive growth. The invasion phenotype was assayed for genetic interactions among 133 genetic perturbations, including gene deletions, plasmid-borne high-copy gene insertions, and dominant negative mutations. The invasion network of the perturbed genes exhibited 41 of the 45 possible interaction inequalities, which were classified by the authors into nine modes of genetic interaction based on classical genetics: *epistatic*, *conditional*, *suppressive*, *single-nonmonotonic*, *additive*, *synthetic*, *asynthetic*, *double-nonmonotonic*, and *non-interactive*. The first four are inherently asymmetric and thus were assigned directionality.

The second data set is from a study by St Onge, et al. of genetic interactions between genes that impart resistance to the DNA-damaging agent methyl methanesulfonate (MMS) [6]. Sensitivity to MMS was assayed in terms of growth rate in the presence of MMS for all pair-wise combinations of deletions for 26 genes. Genetic interactions were initially classified as aggravating, alleviating, or neutral interactions depending on the double-mutant growth rates relative to the two single mutants. Through further analysis the alleviating interactions were sub-classified into five rules of interaction based on models of pathway ordering and protein co-function, four of which were assigned directionality [6]. To explore alternative classifications we converted the data into phenotype inequalities (Materials and Methods), which showed that the MMS-growth network exhibited 10 of the possible 45 interactions.

### Context-dependent complexity

To optimally classify the inequalities by maximizing complexity, we need to define a computable complexity measure for the networks generated by each classification scheme. Because it is context-dependent, the optimal classification scheme will likely vary with the data set being analyzed due to differences in experimental design and the biological system under study. We will therefore use a measure of network complexity that scores networks based on the interrelationships between all elements in the system – true context-dependence. This measure is designed to penalize both information redundancy and randomness, both of which depend on the global network [17]. In genetic interaction analysis, a gene that completely repeats the interaction patters of another gene will not significantly increase the complexity of the system. Likewise, a gene with a perfectly novel interaction pattern will not increase complexity because it adds no information that can be contextualized. Thus networks with a balance between common information and novel information will score higher than those with an excess of redundant or random content.

Calculation of the complexity involves computing both the information content of each element and the mutual information between each pair of elements (Materials and Methods). In previous work we refer to this as a *context-dependent complexity* measure [17], represent this quantity as Ψ, and have normalized it to range from zero (no complexity, e.g. a sequence of random bit strings, or a sequence of identical bit strings) to one (maximal set complexity).

Based on our previous work on the quantitative approach to biological information [5], we conjecture that the classification scheme that maximizes Ψ is the most biologically informative. We note that a “perfect” score of Ψ = 1 is a theoretical bound, and unlikely to be attained for any finite and experimentally-generated data set.

We first assessed the complexity of the invasion and MMS-growth networks using the classifications schemes defined in the original publications [5],[6]. Although many pair-wise combinations of mutations were not assayed in the invasion network study, the single-node and mutual information values were nevertheless calculable for all pairs based on the available data. Thus, we can compute the complexity Ψ, although we note that calculations for networks in which all pair-wise perturbations have been performed are likely to be more statistically robust. However, the larger the network is, with more interactions, the better classifications are generated due to the higher sample size. Results for the published networks are shown in Table 1. For comparison, we also derived interaction networks using the classification scheme from the other work (swapped rules): (i) the invasion network based on the tri-modal scheme, and (ii) the MMS-growth network using the inequality-based nine-mode scheme. Interestingly, in both cases the originally published networks had higher complexity than networks derived from the alternate, swapped rules (Table 1).

**Table 1:** Complexity and number of biological statements obtained for the two genetic interaction networks for various interaction classifications.

*Classification Scheme*	*Invasion*		*MMS-Growth*	
	Ψ	*Statements*	Ψ	*Statements*
Drees, et al. [5]	0.57	68	0.27	41
Segré, et al. [13]	0.52	60	0.32	23
St Onge, et al. [6] *	-	-	0.16	9
Maximal Ψ	0.79	93	0.62	43

*: Subclassification of alleviating interactions could not be performed for the invasion network since this scheme does not have rules to classify every inequality in the invasion data.

The first three classification schemes are from the publications cited. Optimized classifications were determined from unsupervised maximization of complexity Ψ, which has a theoretical maximum of 1. The optimized classification scheme for the invasion data was the greatest found via sampling, whereas the optimal MMS-growth network is the network of absolute maximum complexity, found by exhaustive calculation.

### Unsupervised classification of interactions

The use of the complexity measure, Ψ, allows not only the assessment of classification schemes based on prior knowledge, but also a means to find a classification scheme that maximizes this quantity. Each classification scheme corresponds to a genetic interaction network, and we propose that the network of maximal complexity Ψ corresponds to the network that represents most of the biological information in the interaction data. Thus, we systematically searched for networks with the greatest Ψ for both genetic interaction data sets.

The starting point of the search was the list of pair-wise interaction inequalities, described above, for each network. We grouped these inequalities in all possible ways, ranging from all inequalities in one rule set (corresponding to a trivial network with an identical edge for every experimentally tested pair) to a separate rule for each inequality – most of which occur multiple times in the data. For unsupervised classification we did not assign interactions directionality because of the ambiguities involved in such definitions, for although in some cases it is clear that one mutation can be seen as acting on another (e.g. suppression) in many non-symmetric interactions it is unclear which mutant is causal (e.g. partial suppression versus conditionality [5]). Furthermore, the directionality of the underlying molecular action is often ambiguous. For example, suppression can indicate either molecular bypass or counteraction. Variants of our scheme can take edge directionality into account (e.g., this was done for the published networks in Table 1).

**Figure 1:**
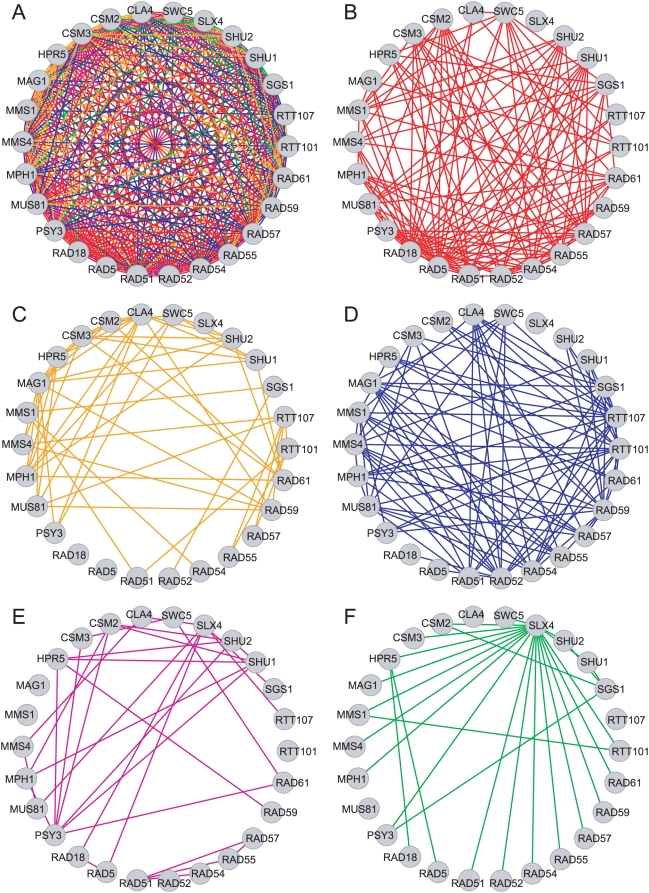
MMS-growth network with maximal set complexity, Ψ. (A) is the complete network. Sub-networks of relationships shown in Table 2 for (B) Rule 1, (C) Rule 2, (D) Rule 3, (E) Rule 4, and (F) Rule 5. The same color codes are used in Figure 3.

The MMS-growth network exhibited only 10 interaction inequalities among the 26 genes, which implies 115,974 possible classifications. Although this is a large number it is amenable to computing the complexity, Ψ, of every possible classification scheme. This was possible due to the relatively small network size, which allowed quick calculation of pair-wise mutual information. We were thus able to determine the optimal classification scheme and know absolutely the maximal complexity score, which was 0.62 (Table 1). The optimal scheme classified the ten inequalities into five rules. These rules are listed in Table 2 and the corresponding network is shown in Figure 1.

**Table 2:** Rules for maximal complexity in the MMS-growth network.

*Rule*	*Rule Frequency*	*Inequalities*	*Inequality Frequency*	*Drees Interpretation*
1	120	*P_AB_ = P_A_<P_B_<P_WT_*	120	epistatic
2	55	*P_AB_<P_A_ = P_B_<P_WT_*	55	additive
3	92	*P_AB_<P_A_<P_B_<P_WT_*	92	additive
4	30	*P_AB_ = P_A_ = P_B_<P_WT_*	24	asynthetic
		*P_AB_ = P_A_<P_B_ = P_WT_*	6	non-interactive
5	26	*P_AB_<P_A_<P_B_ = P_WT_*	14	conditional
		*P_A_<P_AB_ = P_B_<P_WT_*	4	epistatic
		*P_A_<P_AB_<P_B_<P_WT_*	4	single-nonmonotonic
		*P_AB_ = P_A_ = P_B_ = P_WT_*	3	non-interactive
		*P_AB_<P_A_ = P_B_ = P_WT_*	1	synthetic

Frequencies refer to the number of occurrences of the rule in the full network of 323 interactions, and classical interpretations follow Drees et al. [5].

The invasion network contains 41 different phenotype inequalities. The associated search space is much too large for an explicit assessment of every possible classification. To find high-complexity classifications we therefore used a bootstrap algorithm (Materials and Methods). While this procedure cannot ensure that the true maximal complexity classification will be found, we were able to regularly generate classifications with Ψ>0.75, a significant improvement over the published classifications [5]. The final classification scheme corresponded to Ψ = 0.79 (Table 1), which we estimate to be the optimal classification given this data set (the theoretical maximum of Ψ = 1 is not necessarily attainable by any finite, experimentally-generated data set). This classification scheme segregated the interaction inequalities into five classes as shown in Table 3, generating a network with five rules of genetic interaction (consider these as edge types).

**Table 3:** Rules for high complexity in the invasion network.

*Rule*	*Rule Frequency*	*Inequalities*	*Inequality Frequency*	*Drees Interpretation*
1	312	*P_A_<P_WT_ = P_B_ = P_AB_*	146	suppression
		*P_A_ = P_AB_<P_WT_<P_B_*	79	epistatic
		*P_AB_<P_A_<P_WT_ = P_B_*	38	conditional
		*P_AB_<P_WT_ = P_A_<P_B_*	17	conditional
		*P_AB_<P_A_<P_WT_<P_B_*	14	single-nonmonotonic
		*P_WT_<P_A_ = P_B_ = P_AB_*	8	asynthetic
		*P_WT_<P_AB_<P_A_ = P_B_*	4	double-nonmonotonic
		*P_WT_ = P_AB_<P_A_<P_B_*	4	double-nonmonotonic
		*P_WT_<P_A_<P_B_ = P_AB_*	2	epistatic
2	325	*P_A_ = P_AB_<P_B_<P_WT_*	97	epistatic
		*P_A_ = P_AB_ = P_WT_<P_B_*	56	suppression
		*P_A_<P_AB_<P_B_<P_WT_*	50	single-nonmonotonic
		*P_A_<P_WT_<P_B_ = P_AB_*	47	epistatic
		*P_A_<P_WT_ = P_B_<P_AB_*	41	conditional
		*P_WT_ = P_A_ = P_B_<P_AB_*	29	synthetic
		*P_AB_<P_WT_<P_A_<P_B_*	4	double-nonmonotonic
		*P_WT_<P_A_ = P_B_<P_AB_*	1	additive
3	398	*P_A_ = P_AB_<P_WT_ = P_B_*	143	non-interactive
		*P_A_ = P_B_ = P_AB_<P_WT_*	103	asynthetic
		*P_A_<P_B_ = P_AB_<P_WT_*	38	epistatic
		*P_AB_<P_WT_ = P_A_ = P_B_*	33	synthetic
		*P_WT_ = P_A_<P_B_ = P_AB_*	32	non-interactive
		*P_A_<P_B_<P_WT_ = P_AB_*	14	double-nonmonotonic
		*P_A_ = P_B_<P_WT_ = P_AB_*	13	double-nonmonotonic
		*P_WT_ = P_A_<P_B_<P_AB_*	11	conditional
		*P_WT_<P_A_ = P_AB_<P_B_*	8	epistatic
		*P_WT_<P_AB_<P_A_<P_B_*	2	double-nonmonotonic
		*P_A_ = P_B_<P_AB_<P_WT_*	1	double-nonmonotonic
4	356	*P_A_<P_WT_<P_AB_<P_B_*	176	additive
		*P_A_<P_WT_ = P_AB_<P_B_*	105	additive
		*P_WT_ = P_A_<P_AB_<P_B_*	67	conditional
		*P_A_ = P_B_<P_WT_<P_AB_*	8	double-nonmonotonic
5	418	*P_WT_ = P_A_ = P_B_ = P_AB_*	268	non-interactive
		*P_A_<P_AB_<P_WT_ = P_B_*	72	conditional
		*P_A_<P_AB_<P_WT_<P_B_*	40	additive
		*P_AB_<P_A_ = P_B_<P_WT_*	19	additive
		*P_A_<P_WT_<P_B_<P_AB_*	11	single-nonmonotonic
		*P_AB_<P_A_<P_B_<P_WT_*	5	additive
		*P_WT_<P_A_<P_B_<P_AB_*	1	additive
		*P_A_<P_B_<P_WT_<P_AB_*	1	double-nonmonotonic
		*P_WT_ = P_AB_<P_A_ = P_B_*	1	double-nonmonotonic

Frequencies refer to the number of occurrences of the rule in the full network of 1809 interactions, and classical interpretations follow Drees et al. [5].

### Association of Genetic Perturbations with Biological Functions

The power of our approach is that classifying genetic interactions based on maximization of complexity, Ψ, selects the most informative network based on the data alone. The underlying assumption is that the maximally informative representation of the data best corresponds to the genetic interaction “rules” among the perturbations of the evolved biological system at hand, without using any prior, received knowledge of the biology in the maximization procedure. However, we need to define the information content of the network in some independent way to validate this claim.

Most yeast genes have now been annotated for biological function. We can use this prior knowledge after the fact to validate the complexity-based optimization. To do this, we need to define a quantifiable measure of the biological information that can be extracted from a given genetic network using prior knowledge. Although both the genetic interaction data and the annotations contain noise, we can find statistically unlikely occurrences of this kind of relationship, which we call a *biological statement*: a particular gene interacting in a single rule with multiple genes annotated with a single biological function (Materials and Methods; [5]). The result was a computer-generated list of *biological statements* relating genes, interaction modes, and target annotations, with entries such as: “A deletion of *RAD52* interacts *via* Rule 1 with deletion mutations of non-recombinatorial repair genes. (p = 0.0019).” The number of such existing statements will be highly sensitive to the interaction rules in the network, and thus can serve as a measure of how informative each classification scheme is in a biological sense.

**Figure 2:**
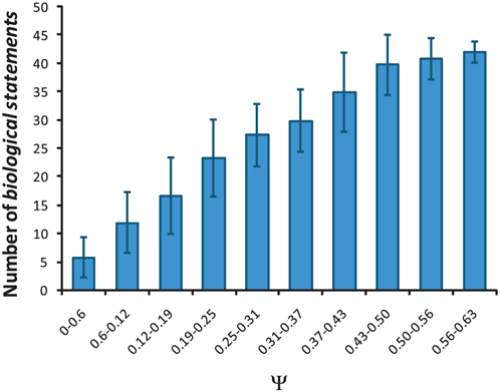
Biological information as a function of set complexity Ψ in the MMS-growth networks. Average number of *biological statements* (significance *P*<0.01) for binned complexity calculated from all possible networks. Error bars denote the standard deviation of binned data points.

A list of biological statements was generated for every possible MSS-growth network and every sampled invasion network. The complete list for the maximally complex MMS-growth network is shown in Table 4. Considering all possible MMS-growth networks, we found a strong correlation between the number of statements and the information score Ψ (*r* = 0.80). The 115,974 data points (one for each network) are summarized in the histogram in Figure 2, which shows the mean number of biological statements as a function of binned Ψ values. There is a clear trend that a greater information score corresponds to a greater number of biological statements extracted. We stress that these two quantities are entirely independent and there is no *a priori* reason for such correlation. A similar correlation was observed in the invasive growth network, although we could only sample a small subset of random classifications given the greater size of the network. This finding is strong evidence that maximizing the context-based information measure Ψ is a good strategy for inferring information about real biological organization. This conclusion is strengthened when we examine the inferred networks and the implied biological organization, as discussed below. Our mathematical measure appears to reflect the real biological organization represented in the data.

**Table 4:** Biological statements extracted from the maximally complex MMS-growth network.

*Deletion of gene*	*interacts via*	*with deletions of genes with GO annotation*	*P*
SGS1	Rule 5	error-free DNA repair	7.91E-05
SWC5	Rule 2	error-free DNA repair	0.00040
RAD51	Rule 4	heteroduplex formation	0.00043
CLA4	Rule 3	developmental process	0.00047
PSY3	Rule 1	meiosis I	0.00047
CLA4	Rule 3	DNA recombination	0.00098
CSM2	Rule 1	meiosis I	0.0012
PSY3	Rule 3	negative regulation of transposition, RNA-mediated	0.0017
MPH1	Rule 4	error-free DNA repair	0.0017
CSM2	Rule 4	error-free DNA repair	0.0017
SHU2	Rule 4	error-free DNA repair	0.0020
HPR5	Rule 1	mitotic recombination	0.0022
CLA4	Rule 3	reproductive developmental process	0.0024
PSY3	Rule 1	reproductive developmental process	0.0024
SHU1	Rule 1	reproductive developmental process	0.0024
MAG1	Rule 3	reproductive developmental process	0.0024
RAD52	Rule 1	double-strand break repair via single-strand annealing	0.0026
HPR5	Rule 1	cellular component organization and biogenesis	0.0026
MPH1	Rule 1	heteroduplex formation	0.0028
CLA4	Rule 3	mitotic recombination	0.0029
MAG1	Rule 3	mitotic recombination	0.0029
SHU2	Rule 1	meiosis I	0.0030
HPR5	Rule 3	negative regulation of transposition, RNA-mediated	0.0043
SHU1	Rule 4	error-free DNA repair	0.0043
RAD59	Rule 1	postreplication repair	0.0043
RAD52	Rule 1	non-recombinational repair	0.0044
CSM2	Rule 1	reproductive developmental process	0.0047
HPR5	Rule 1	reproductive developmental process	0.0055
HPR5	Rule 4	error-free DNA repair	0.0067
RTT101	Rule 2	heteroduplex formation	0.0067
MPH1	Rule 3	DNA recombination	0.0071
MMS1	Rule 2	telomere maintenance via recombination	0.0087
HPR5	Rule 1	meiotic DNA recombinase assembly	0.0087
RAD51	Rule 4	double-strand break repair via single-strand annealing	0.0087
MMS4	Rule 3	reproductive developmental process	0.0087
RTT107	Rule 2	DNA recombination	0.0097
CLA4	Rule 3	heteroduplex formation	0.0100
CSM3	Rule 1	heteroduplex formation	0.0100
PSY3	Rule 1	heteroduplex formation	0.0100
SHU1	Rule 1	heteroduplex formation	0.0100
MAG1	Rule 3	heteroduplex formation	0.0100
MUS81	Rule 3	heteroduplex formation	0.0100
MUS81	Rule 1	error-free DNA repair	0.0100

### Mutual Information Networks

**Figure 3:**
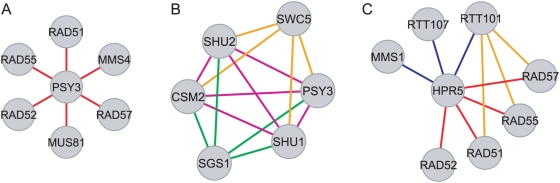
Examples of biological information extracted from the maximally complex MMS-growth network. (A) Deletion of *PSY3* interacts via Rule 1 (red edges) with meiotic recombination gene deletions. (B) Deletion of *SGS1* interacts via Rule 5 (green edges) with four error-free DNA repair gene deletions. Deletion of *SWC5* interacts with the same genes via Rule 2 (orange edges). These four genes interact via Rule 4 (violet edges), significantly for *CSM2* and *SHU2* deletions. (C) Deletion of *HPR5* interacts via Rule 3 (blue edges) with genes involved in negative regulation of DNA transposition and via Rule 1 (red edges) to genes involved in gene conversion at mating-type locus. Deletion of *RTT101* interacts via Rule 2 (orange edges) with heteroduplex formation genes.

**Figure 4:**
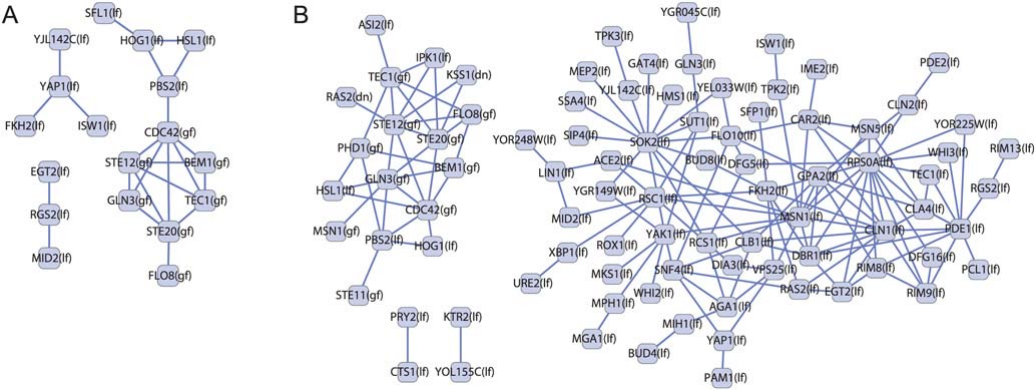
Networks of mutual information for yeast invasion data. Nodes represent alleles and edges represent significant mutual information between the connected alleles. (A) Mutual information network obtained using the classification scheme of Drees, et al, showing all pairs of significance *p*<0.001 [5]. (B) Mutual information network obtained using the maximally complex classification scheme on the same data, showing all pairs of significance *p*<0.0001. The maximally complex classification scheme produces more pairs and higher significance.

While biological statements characterize local network properties and define functional subnetworks (Figure 3), global patterns in genetic interaction networks can also reveal underlying biology. In particular, pairs of alleles often show a high degree of mutual information with common interaction partners, such that knowing the interactions of one allele may allow one to know the interactions of another. This pair-wise property is quantified by the mutual information scores used to compute the context-dependent complexity metric, Ψ (Materials and Methods, Eqn. 2). We identified pairs of alleles with statistically significant mutual information (Materials and Methods). These pairs were mapped in mutual information networks (Figure 4). Clusters or cliques of genes in a mutual information network identify genes with similar effects on biological processes, often corresponding to specific modules [5]. Therefore a larger number of mutually informative pairs corresponds to more comprehensive module mapping.

We analyzed the yeast invasion network for significant mutual information (Materials and Methods). We were unable to similarly randomize the MMS sensitivity network due to a limited diversity of interaction types. Drees, *et al* identified 23 allele pairs with significant (*P*<0.001) mutual information using their edge classification scheme (Figure 4A) [5]. Our maximally complex classification scheme allowed a more stringent P-value cutoff. We identified 159 allele pairs with significant (*P*<0.0001) mutual information (Figure 4B and Table S1), compared to 15 allele pairs of this higher significance in the Drees network. Inspection of classification schemes with intermediate complexity scores showed that the number of mutually informative alleles generally increases with Ψ (data not shown), similar to the trend observed for biological statements (Figure 2).

The mutual information network arising from the maximally complex classification scheme reveals two major subnetworks, or modules, of connected components (Figure 4B). The first, containing 17 alleles, is statistically enriched in invasive growth and cell-surface receptor linked signal transduction genes (Table S2). The majority of these genes are components of the MAP kinase signaling pathway that promotes invasive filamentous-form growth (fMAPK pathway) [18]. The second, larger module comprises 66 alleles and has no homolog in the mutual information network obtained from the Drees classification scheme (Figure 4A). It is enriched in DNA-dependent transcription and cAMP-mediated signaling genes (Table S2). The fMAPK and cAMP signaling modules are known to be major determinants of the cellular switch to filamentous-form growth [19].

## Discussion

### Set Complexity and Biological Information

In deriving networks automatically based on maximal set complexity we were able to both recover previous insights and discover new biological information. The sub-networks in Figure 3 illustrate examples of *biological statements* from the maximally complex MMS-growth network (Table 4). Although detailed interpretations of the molecular biology underlying this is beyond the scope of this study, we find that the network derived from maximization of set complexity reflects novel and diverse biological relationships between genes in the network. Figure 3A shows that the gene *PSY3* interacts functionally via Rule 1 with six meiotic recombination genes, a conclusion not obtained in the supervised analysis published previously. The sub-network in Figure 3B illustrates a set of relationships involving the DNA repair genes *CSM2*, *SHU1*, *SHU2*, and *PSY3*, which generated four significant biological statements (Table 4). *SWC5* interacts with these genes *via* Rule 2, whereas *SGS1* interacts *via* Rule 5. Furthermore, the DNA repair genes themselves form a clique of Rule 4 interactions, which is statistically significant for *CSM2* and *SHU2*. The latter three statements were discussed in the original analysis [6], to which our complexity-based analysis adds an additional piece of information. Finally, we show in Figure 3C that *HPR5* interacts specifically *via* Rule 3 with genes determining the negative regulation of DNA transposition, *MMS1*, *RTT101*, and *RTT107*, and *via* Rule 1 (epistasis) to four genes involved in gene conversion at mating-type locus. This sub-network also illustrates the Rule-2 interactions between *RTT101* and three hetero-duplex formation genes. These relationships were not obtained in the previous analysis and are a result of deriving the network based on maximization of set complexity.

Unsupervised classification of the invasion network generated a network of highest complexity score with five rules of genetic interaction. Because interaction data were generated for a larger number of gene pairs (1809 interactions tested between 133 gene mutations) than in the MMS-growth data, many more inequalities were observed (41 compared to ten in MMS-growth). These inequalities were grouped into five rules, comprising between five and 11 inequalities each. These rules are listed and are directly compared with the original classifications of Drees, et al. [5] in Table 3. In Drees, et al. many of the inequalities were assigned rules based on classical genetics; some were unfamiliar and grouped based on mathematical relationships. For example, ten of the 41 inequalities were grouped together as *double-nonmonotonic*, an interaction rule without an established biological interpretation. Our complexity-based analysis distributed these ten inequalities across all five rules, grouping some of them with primarily additive interactions (Rule 5) while others share a rule with epistatic and suppressive interactions (Rule 1).

**Figure 5:**
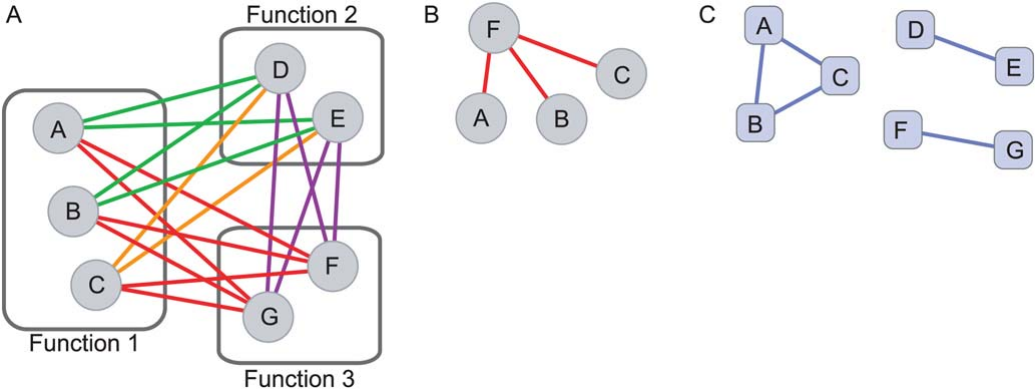
Network modularity of genetic interactions. (A) A simple, hypothetical genetic interaction network of seven genes with three biological functions. (B) An example biological statement inferred from the genetic interaction network, establishing a coherent interaction rule between gene perturbation F and Function 1. (C) Inferred mutual information network that exhibits the functional modularity of the genetic network.

Overall, the rules derived for the invasion data often do not correspond to those in pathway-oriented analysis [5]. Interpretation of these rules therefore requires further analysis, keeping in mind that more than one mutation type was used to generate the invasion data (gene deletions, gains-of-function, etc.). However, the results might seem less perplexing in light of the fact that varying the allele forms of the same two genes can result in different genetic interaction inequalities. Furthermore, genes playing different roles (activation, inhibition, etc.) in the same functional module might interact in different modes with perturbations of genes in a different module. The grouping of interactions into rules that maximize set complexity apparently groups the interactions into coherent rules relating genes in different modules, rather than basing interaction rules on each pair of genes individually. This concept is illustrated in Figure 5. In this light, the classifications derived from the complexity metric are interpreted as the rules that govern how genes are organized into functional groups, taking into account the full content (and limitations) of the analyzed data set. This can be contrasted with the pathway analysis of genetic interactions, in which the rules are interpreted in terms of information flow through individual gene pairs.

Network analysis results support this interpretation. As with the MMS-growth network, the invasion network resulting from this classification scheme resulted in a marked increase in *biological statements* obtained from the network (Table 1). Thus the network is a promising candidate for analysis with computational tools that search for patterns and motifs in complex data [20]. The statements listed in Tables 4 and 5 and the corresponding example sub-networks in Figure 3 involve network motifs and architecture that can be systematically interpreted for biological meaning by higher level, unsupervised analyses [20]–[23]. The quality of such tools is critically dependent on the global network used as input, and our results strongly suggest that networks derived from maximization of set complexity are the optimal inputs for information-based network analyses.

**Table 5:** Biological statements extracted from the high-complexity invasion network.

*Mutation of*	*interacts via*	*with mutations of genes with GO annotation*	*P*
HOG1	Rule 4	invasive growth in response to glucose limitation	0.00027
TEC1	Rule 2	regulation of cellular process	0.00031
STE11	Rule 3	organelle organization and biogenesis	0.00053
HOG1	Rule 4	positive regulation of biological process	0.00064
XBP1	Rule 5	reproduction	0.00070
ROX1	Rule 5	conjugation with cellular fusion	0.0010
HOG1	Rule 4	positive regulation of transcription from RNA polymerase II promoter	0.0011
RAS2	Rule 2	positive regulation of biological process	0.0011
SPO12	Rule 5	reproduction	0.0012
MSN1	Rule 1	cell surface receptor linked signal transduction	0.0014
KSS1	Rule 2	positive regulation of catalytic activity	0.0014
KSS1	Rule 4	regulation of nucleobase, nucleoside, nucleotide and nucleic acid metabolic process	0.0014
HSL1	Rule 1	cell wall organization and biogenesis	0.0014
HOG1	Rule 4	conjugation with cellular fusion	0.0015
CDC42	Rule 4	positive regulation of metabolic process	0.0015
SRL1	Rule 3	conjugation with cellular fusion	0.0015
MSN1	Rule 1	positive regulation of catalytic activity	0.0018
COD4	Rule 5	reproduction	0.0019
RAS2	Rule 4	regulation of nucleobase, nucleoside, nucleotide and nucleic acid metabolic process	0.0019
HOG1	Rule 4	response to pheromone	0.0021
HOG1	Rule 4	pheromone-dependent signal transduction during conjugation with cellular fusion	0.0021
RAS2	Rule 2	response to pheromone	0.0022
GLN3	Rule 2	protein targeting	0.0023
KSS1	Rule 2	intracellular signaling cascade	0.0023
URE2	Rule 3	reproduction	0.0025
URE2	Rule 3	response to pheromone	0.0025
YPS1	Rule 3	G-protein coupled receptor protein signaling pathway	0.0025
KTR2	Rule 3	G-protein coupled receptor protein signaling pathway	0.0025
TEC1	Rule 2	positive regulation of biological process	0.0025
DIG2	Rule 2	osmosensory signaling pathway	0.0027
PBS2	Rule 4	replicative cell aging	0.0031
XBP1	Rule 5	cell communication	0.0031
XBP1	Rule 5	response to chemical stimulus	0.0031
CDC42	Rule 4	regulation of transcription, DNA-dependent	0.0032
YPS1	Rule 3	filamentous growth	0.0038
STE12	Rule 4	positive regulation of metabolic process	0.0038
STE20	Rule 4	positive regulation of metabolic process	0.0038
DSE1	Rule 5	cellular component organization and biogenesis	0.0039
TEC1	Rule 4	regulation of nucleobase, nucleoside, nucleotide and nucleic acid metabolic process	0.0039
HSL1	Rule 5	pheromone-dependent signal transduction during conjugation with cellular fusion	0.0040
STE12	Rule 3	cell wall organization and biogenesis	0.0040
STE20	Rule 3	cell wall organization and biogenesis	0.0040
PRY2	Rule 2	osmosensory signaling pathway	0.0041
IPK1	Rule 4	invasive growth in response to glucose limitation	0.0043
KSS1	Rule 2	protein amino acid phosphorylation	0.0043
YPL114W	Rule 5	developmental process	0.0045
HOG1	Rule 5	protein targeting	0.0048
PDE2	Rule 3	intracellular signaling cascade	0.0048
DIG2	Rule 2	response to chemical stimulus	0.0048
ROX1	Rule 5	response to pheromone during conjugation with cellular fusion	0.0048
TPK1	Rule 2	cell surface receptor linked signal transduction	0.0049
YJL017W	Rule 5	establishment of cell polarity	0.0049
YJL017W	Rule 1	positive regulation of catalytic activity	0.0049
MIH1	Rule 5	cell communication	0.0051
STE20	Rule 2	growth	0.0052
RSR1	Rule 1	sporulation	0.0054
STE20	Rule 2	filamentous growth	0.0055
MSN1	Rule 2	M phase of mitotic cell cycle	0.0055
MSN1	Rule 2	conjugation with cellular fusion	0.0055
RSR1	Rule 3	cellular metabolic process	0.0057
DBR1	Rule 2	mitotic cell cycle	0.0059
TEC1	Rule 4	negative regulation of nucleobase, nucleoside, nucleotide and nucleic acid metabolic process	0.0060
MSN1	Rule 2	invasive growth in response to glucose limitation	0.0062
SRL1	Rule 3	response to pheromone during conjugation with cellular fusion	0.0065
MSN1	Rule 3	cell communication	0.0068
TEC1	Rule 2	positive regulation of metabolic process	0.0071
CTS1	Rule 2	osmosensory signaling pathway	0.0071
PHD1	Rule 5	protein localization	0.0073
RIM13	Rule 5	conjugation with cellular fusion	0.0076
RIM13	Rule 5	invasive growth in response to glucose limitation	0.0076
CLN3	Rule 3	response to pheromone	0.0076
DIA3	Rule 5	response to pheromone	0.0076
BUD4	Rule 1	regulation of molecular function	0.0077
IME2	Rule 3	G-protein coupled receptor protein signaling pathway	0.0077
PAM1	Rule 3	cell morphogenesis	0.0077
KSS1	Rule 1	biopolymer metabolic process	0.0081
GPR1	Rule 1	response to pheromone	0.0082
BEM1	Rule 1	nitrogen utilization	0.0085
STE12	Rule 1	intracellular signaling cascade	0.0087
STE12	Rule 3	nitrogen utilization	0.0090
IPK1	Rule 4	conjugation with cellular fusion	0.0090
CDC42	Rule 3	cell wall organization and biogenesis	0.0090
URE2	Rule 3	signal transduction	0.0090
YPS1	Rule 3	reproduction	0.0090
YPS1	Rule 3	invasive growth in response to glucose limitation	0.0090
YPS1	Rule 3	pseudohyphal growth	0.0090
KTR2	Rule 3	reproduction	0.0090
MSN1	Rule 1	G-protein coupled receptor protein signaling pathway	0.0092
RSR1	Rule 3	cellular macromolecule metabolic process	0.0093
CDC42	Rule 4	regulation of transcription from RNA polymerase II promoter	0.0093
FLO8	Rule 5	filamentous growth	0.0094
GLN3	Rule 2	macromolecule metabolic process	0.0095
IPK1	Rule 3	cell communication	0.0095

The demonstrated correlation between set complexity, Ψ, and the number of network-generated *biological statements* is strong evidence that the former is a sound basis for analyzing genetic interaction data sets. Furthermore, we found that classification schemes that produce networks of greater complexity also generate larger and more informative mutual information networks. Yeast invasion network alleles were separated into two clusters corresponding to genes in the Kss1-based MAP-kinase and cAMP-mediated signaling modules (Figure 4B) [18],[24]. Co-activation of these two signaling modules is known to play a major role in initiating yeast invasive growth, however many genes with invasive-growth phenotypes have not yet been associated with these (or other invasion-related) modules. Placement in the mutual information network suggests module associations for some of these genes. For example, high throughput assays have found that deletion of the gene *VPS25* causes diminished pseudohyphal [25] and invasive growth [5] but its relevant function is unclear. Due to its position in the largest mutual information cluster, our analysis suggests the defects are due to Vps25 affecting cAMP signaling during invasive growth. Furthermore, although we did not detect any in this data set, additional large mutual information clusters might implicate other modules affecting a phenotype.

We note that although the complexity maximization procedure favors schemes that increase pair-wise mutual information, the procedure does not formally maximize this quantity. An overabundance of mutual information can hide systematic distinctions between allele pairs and thus corresponds to a loss of complexity in the network. Instead, the procedure attempts to find the classification scheme which best distributes the mutual information in a given data set, effectively determining the optimal resolution of pair-wise mutual information. This is evident in the invasion network analysis (Figure 4B), in which over half of the tested alleles (87 out of 133) were found to have at least one mutually informative partner and MAPK and cAMP signaling module elements were accurately separated.

We stress that the interaction classifications (rules) were selected only by maximizing the complexity measure, Ψ – based on the data alone, and without using any prior knowledge of the biology under study. Gene annotations and mutual information networks were used subsequent to the rule choice to validate the biological relevance of the optimized networks.

### The Set Complexity Metric Ψ

**Figure 6:**
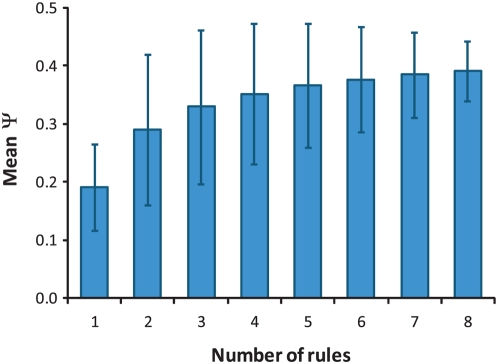
Set complexity Ψ as a function of the number of interaction rules in the MMS-growth networks. Average complexity as a function of number of rules for all possible networks. Error bars denote the standard deviation of binned data points.

**Figure 7:**
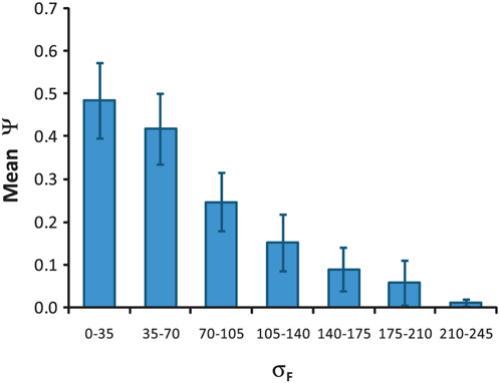
Set complexity Ψ as a function of the standard deviation of interaction rule frequencies in the MMS-growth networks. Average complexity for binned standard deviations of rule frequencies calculated from all possible networks. Error bars denote the standard deviation of binned data points.

Examining the genetic interaction networks generated by each classification scheme reveals some general properties of the set complexity measure Ψ. While a greater number of interaction rules in a given classification scheme tended to generate a larger Ψ (Pearson *r* = 0.18), this association was weak and a large range of complexity values were found for every number of rules (Figure 6). A stronger association was found between uniformity in the frequencies of each interaction rule in the network and complexity Ψ. This was quantified by computing the frequency of each interaction rule in a given network, then taking the standard deviation σ_F_ of the list of frequencies. Thus a network with a wide variation in number of occurrences for different interaction modes will have a large σ_F_. We computed σ_F_ for every possible classification of the MMS-growth data and found a very strong anti-correlation between σ_F_ and Ψ (Pearson *r* = −0.85). This is shown in Figure 7, which plots the average Ψ complexity for networks with specified ranges in σ_F_. This association originates in the context dependence of the complexity measure, which generates scores based not only on the information content of each node, but the sum content of all mutual information in the network (Materials and Methods, Eqn. 3). In general, interaction rules that are infrequent (relative to other rules in the network) cannot be associated with a functional role, since extraction of *biological statements* or other conclusions is often statistically insignificant.

This property of our complexity measure explains the reduction in complexity when the MMS-growth network based on the tri-modal classification scheme (aggravating, neutral, alleviating) is sub-classified into six modes (aggravating, neutral, and four classes of alleviating) based on MMS-sensitivity (Table 1) [6]. Three of the alleviating subclasses of interaction appear fewer than 10 times each in the global network, out of 323 total edges. Although prior information could be used to interpret these interactions [6], it is unclear how any meaning can be ascribed to them from the data alone. Similar difficulties were seen in the invasion network, in which the greatest complexity scores occurred for five or six interaction rules, suggesting the original classification scheme into 9 rules over-specified the network. On the other hand, interaction rules that occur too commonly fail to clearly attribute any biological meaning to a specific rule, since they are weakly associated with many different functions. For example, simply classifying the invasion network into two classes – “interacting” and “non-interacting” – generated a set complexity score of 0.26 and relatively few *biological statements*. Similarly classifying the MMS-growth network into “neutral” and “non-neutral” interaction rules generated a complexity of 0.22 and only five *biological statements*. These findings demonstrate the importance of analyzing complexity in a context-dependent way, by determining the meaning of any single interaction from how it relates to all other interactions in the network. It appears that maximizing Ψ inherently avoids over-fitting, and balances the two effects.

However, we also note that uniformity in rule frequencies (low σ_F_) is an indirectly contributing factor in complexity. Our definition of complexity (Materials and Methods, Eqn. 3) fundamentally depends on the amount and distribution of mutual information in the network [17]. Thus uniformity in rule frequencies can be viewed as a necessary condition of mutual information generation, but it alone is not sufficient. Mutual information must be inherent in the data itself in order to obtain networks with high complexity. Thus, although the maximally complex MMS-growth network (σ_F_ = 40.6) could be made more uniform in rule frequencies by merging the less frequent rules into a single rule (e.g. Rules 2, 4, and 5 in Table 2), such classification schemes have a lower complexity for this data set. Similarly, although the rules in the invasion network occur with fairly uniform frequencies, none of the many schemes with greater uniformity was found to generate a higher complexity score. The results suggest that the higher-complexity schemes of lesser uniformity detect the modular organization of the underlying molecular network.

It is important to keep in mind that the specific mathematical form of Ψ is somewhat arbitrary, being selected as the simplest function of the “universal information distance” [26] that has zeros at 0 and 1. Other functions with this property will have maxima at different values, and we expect they can affect the classifications at a maximum of the modified Ψ. These alternatives need to be explored, but the important point for the present work is that Ψ works well using the simple function.

### Prospects

The results of Table 1 demonstrate that although the published classification schemes generate invasion and MMS-growth networks with substantial complexity, in both cases the interaction data could be reclassified to produce more informative networks. This is true both in terms of the context-dependent complexity Ψ and the number of biological statements extracted. We note that the original classifications of the invasion network [5] scored quite highly, which is probably due to the classifications being carefully based on rules of genetic interaction that geneticists have determined by experience to be biologically informative. Nonetheless, the interaction rules derived from the maximization of set complexity seem to bear little resemblance to these. We propose that the classical genetics rules discover molecular information flow sequences, or pathways, whereas Ψ-based rules detect higher-level relationships between functional network modules. A further implication of this interpretation is that one should not expect that there is any universal classification of genetic interactions into rules. Rather, one should expect that optimal rules will depend on the genes, alleles, modules, networks, organisms, environmental conditions, and phenotypes under examination. This is all the more reason for an unsupervised computable approach to the problem without the need for extensive prior knowledge. In analyzing complex traits in mammals the number and complexity of interactions are likely to increase and gene function annotations are less complete. An automated classification is likely to be the only feasible way of approaching the problem. Additional computational and network analysis will be needed to interpret interaction rules in terms of biological activity.

In this work, we have used only genetic interaction data, processed into phenotype inequalities, as input for the optimization problem. The interactions were therefore groups of inequalities that were classified into sets of interactions called rules. This procedure, however, can be easily expanded to include additional data. Any additional information characterizing the relationship between two genes (including annotation and experimental data) can be appended to the inequality, creating a more complex set of interactions, and the classification can then be redone by maximizing the modified set complexity. For example, measuring a second phenotype will change each gene-pair relationship from one inequality constructed on a linear axis (relating the points *P_WT_*, *P_A_*, etc.) to a two-dimensional relationship in which the values of each mutant strain are plotted relative to an axis for each phenotype (relating the points {*P^1^_WT_*, *P^2^_WT_*}, {*P^1^_A_*, *P^2^_A_*}, etc.). Each additional measurement, such as gene expression data, would add another phenotypic dimension to the relationship. Continued data integration will rapidly render individual gene-pair relationships conceptually intractable, but the relationships would still be amenable to analysis with our complexity measure. Such extensions of the present work are currently being explored with the expectation that the more accurate data (and data types) that are added to the characterization of gene (and gene product) interactions, the more significant biological information can be extracted using a set complexity maximization approach.

## Materials and Methods

### Data Sources

The invasion network was taken directly from Drees et al. [5]. The MMS-growth interactions were derived from data published by St. Onge et al. [6]. Since growth mutations are expected to combine multiplicatively, we log-transformed the fitness values for our linear comparisons. An error model was necessary to order the log-fitness data into phenotype inequalities. From the original analysis we noted that values of *W_XY_−W_X_×W_Y_* greater than 0.078 were uniformly significant, independent of the magnitudes of fitness values. Thus, we assumed an error range of *δ* = 0.039 for each fitness value *W_S_*, where the subscript refers to any strain. We then computed each log-transformed fitness value *p_S_* = log(*W_S_*), and estimated the error to be the mean of adding or subtracting the error and log-transforming, which is *Δ_S_* = 0.5 log((*W_S_*+*δ*)/(*W_S_*−*δ*)). Following the analysis of Drees, et al. [5], we defined each phenotype on the interval *P_S_* = [*p_S_*−*Δ_S_*, *p_S_*+*Δ_S_*] and assumed any two phenotypes to be equivalent if there was any overlap in their intervals [5]. By comparing phenotype intervals we constructed the phenotype inequalities like those shown in Table 2, such as *P_WT_<P_AB_ = P_A_<P_B_*.

### Calculation of Context-Dependent Complexity

While the measure was originally defined in terms of Kolmogorov-Chaitin-Solomonoff complexity [17] it is clear that if the sample space is well defined, as it is here, we can use the Shannon approach of calculating complexities with probabilities [27]. We define the context-dependent, set complexity for a network as follows. For the *i*-th node in a network, we first compute the Shannon information, *K_i_*, based on its interactions with all other nodes. These interactions will fall into different classes, depending on the classification scheme being used. For the *i*-th node, we compute the fraction of nearest neighbors with each class of interaction, denoted *p_i_(a)* for the *a*-th interaction class. Summing over all interactions types yields the single-node complexity:
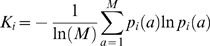
(1)where *M* is the number of interaction classes and the sum is over all interaction classes. The normalization ensures that this quantity is always between 0 and 1. Directionality of interactions was retained where relevant, so that outgoing edges were considered a different interaction type than incoming edges. We next compute the mutual information for every pair of nodes in the network using the Shannon approach. This can be written
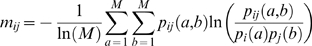
(2)where *p_ij_(a,b)* is the joint probability of node *i* interacting with a third node with rule *a* and node *j* interacting with the same third node with rule *b*. This expression is also normalized to the interval [0,1].

With these normalized quantities we compute the context-dependent complexity of a network with *N* nodes by summing over all node pairs as:
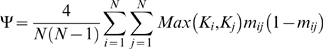
(3)This complexity measure, which has been normalized to yield values between 0 and 1, was derived in Galas, et al. [17]. It is based on the normalized information distance function between two strings as derived by Li, et al. [26], which is a metric satisfying the three criteria of identity, symmetry, and the triangle inequality. This metric is universal in that it discovers all computable similarities between strings [26]. As shown by Galas, et al. [17], a simple relationship between the universal information distance and pair-wise mutual information allows Ψ to be computed with mutual information (Eqn. 2). Although the metric function *m_ij_*(1−*m_ij_*) in the pair-wise sum does not uniquely define a set-dependent complexity measure, it is the simplest form that discounts both redundant (high *m_ij_*) and unrelated (low *m_ij_*) information [17]. In practice, calculations with real data are generally insensitive to the precise form of this metric function. Each genetic interaction network was scored with the set complexity Ψ (Eqn. 3). Different classification schemes of genetic interactions generated variations in the single-node entropy (*K_i_*) and mutual information between node pairs (*m_ij_*), leading to variation in Ψ.

### Bootstrap Algorithm for Unsupervised Classification of the Invasion Network

We first selected six inequalities and constructed the sub-network involving these interactions only. We tested all possible classifications of these six seed interactions (there were 202) and determined the sub-network with maximal Ψ. The classifications in this sub-network were used as a seed. We then built up the full network adding one interaction inequality at a time, assigning it to one of the seed rules or allowing it to start a new rule, with the decision determined by the choice that generated the maximal-complexity network. Once all 41 interaction inequalities were assigned a rule and the full network was determined, we randomly perturbed the classifications in search of a gain in set complexity, Ψ. The perturbations were randomly chosen by: randomly re-assigning rules for one, two, three, or four inequalities; splitting a randomly chosen rule into two rules, with new rule assignments chosen randomly; or merging two randomly selected rules. At least 1000 random perturbations were carried out for roughly 100 bootstrap-generated classifications.

### Genetic Interaction with Biological Processes

In order to measure the biological information that can be extracted from a genetic interaction network, we identified statistically significant correlations between a given gene mutation's interaction rules and mutations of genes involved in a common biological process. The neighbors of every node (gene mutation) in the network were queried for interaction class and Gene Ontology Consortium Database annotations [28]. Likelihood values were computed to find over-represented class-annotation pairings within each set of nearest neighbors, and *P*-values were calculated relative to a cumulative hypergeometric distribution.

Since GO annotations are not independently assigned to genes, we did not choose an arbitrary significance cut and apply a Bonferroni correction. Instead, we empirically determined the maximum *P*-value for a significant biological statement. For the invasion network, we followed the analysis in Drees et al. [5] and applied a significance cut of *P*<0.01 (more stringent than the *P*<0.05 in that work). For the MMS-growth network, we randomly permuted the names of every gene and recomputed the biological statements for networks for sample classifications. This strategy: (i) retains the same genes and gene annotations, thus maintaining the same number of annotations and interdependences between annotations; and (ii) retains the network's topology and edge types (and hence the complexity score) for each classification scheme. Re-computing every classification scheme for each randomization was computationally infeasible, so 1150 classification schemes were computed for each randomized network (every tenth scheme). This produced a representative set of schemes in terms of number and frequency of rules in the network. From 100 such randomizations we determined that a significance cut of *P*<0.01 has a false-positive rate of 8% (eight of every 100 biological statements with *P*<0.01 are probably background noise). We note that our primary use of these statements was to assess the relationship between complexity and biological information rather than their biological content *per se*, and thus we accepted a fairly high error rate in order to have a large sample size.

### Mutual Information Networks

In order to detect global similarity between the interaction patterns of two alleles, we detected nodes with mutual information (Eq. 2) significantly higher than expectation. Significance of mutual information was tested independently for each allele pair by computing the likelihood of obtaining the observed score in randomly permuted data, following the procedure in Drees, *et al* [5]. To remove bias due to our selection of mutant alleles, randomized data were constrained by keeping the wild-type and two single-mutant phenotypes fixed and replacing interaction modes only with modes that are consistent with the observed single-mutant phenotypes. The choice among possible replacement modes was weighted by observed frequency in the entire network. Five thousand randomizations were carried out to determine a mean and standard deviation to characterize the distribution for each tested allele pair. P-values were then calculated as the probability of finding a mutual information score at or above the observed score. In Figure 4A we reproduce the results of Drees *et al*, which used a P-value cutoff of 0.001 [5]. For the maximally complex classification scheme (Figure 4B), we chose a more stringent cutoff of *P*<0.0001. The false discovery rate for the maximally complex classification scheme was estimated empirically from the number of mutually informative gene pairs in 200 randomized networks. For the P-value cutoff of *P*<0.0001, we found a mean number of 3.5 allele pairs (false positives), compared with 159 found in the actual data. Enrichments of Gene Ontology terms in the two major connected components of this network were computed following the method described above in Genetic Interaction with Biological Processes. We were unable to perform the randomization procedure (and hence the analysis) on the MMS sensitivity network due to the frequent occurrence of a unique double-mutant phenotype for a given pair of single-mutant phenotypes, which precluded random permutation of the double-mutant values.

## Supporting Information

Table S1 Supplemental Table S1
(0.03 MB XLS)

Table S2 Supplemental Table S2
(0.02 MB XLS)
